# H3K36 methylation reprograms gene expression to drive early gametocyte development in Plasmodium falciparum

**DOI:** 10.1186/s13072-021-00393-9

**Published:** 2021-04-01

**Authors:** Jessica Connacher, Gabrielle A. Josling, Lindsey M. Orchard, Janette Reader, Manuel Llinás, Lyn-Marié Birkholtz

**Affiliations:** 1 Department of Biochemistry, Genetics and Microbiology, Institute for Sustainable Malaria Control, University of Pretoria, Private Bag x20, Hatfield, 0028 South Africa; 2 Department of Biochemistry & Molecular Biology and the Huck Center for Malaria Research, Pennsylvania State University, University Park, PA 16802 USA; 3 Department of Chemistry, Pennsylvania State University, University Park, PA 16802 USA

**Keywords:** H3K36me2, H3K36me3, Histone, Malaria, *Plasmodium*, Gametocyte, Epigenetics, Histone post-translational modifications, Histone demethylation

## Abstract

**Background:**

The *Plasmodium* sexual gametocyte stages are the only transmissible form of the malaria parasite and are thus responsible for the continued transmission of the disease. Gametocytes undergo extensive functional and morphological changes from commitment to maturity, directed by an equally extensive control program. However, the processes that drive the differentiation and development of the gametocyte post-commitment, remain largely unexplored. A previous study reported enrichment of H3K36 di- and tri-methylated (H3K36me2&3) histones in early-stage gametocytes. Using chromatin immunoprecipitation followed by high-throughput sequencing, we identify a stage-specific association between these repressive histone modifications and transcriptional reprogramming that define a stage II gametocyte transition point.

**Results:**

Here, we show that H3K36me2 and H3K36me3 from stage II gametocytes are associated with repression of genes involved in asexual proliferation and sexual commitment, indicating that H3K36me2&3-mediated repression of such genes is essential to the transition from early gametocyte differentiation to intermediate development. Importantly, we show that the gene encoding the transcription factor AP2-G as commitment master regulator is enriched with H3K36me2&3 and actively repressed in stage II gametocytes, providing the first evidence of *ap2-g* gene repression in post-commitment gametocytes. Lastly, we associate the enhanced potency of the pan-selective Jumonji inhibitor JIB-04 in gametocytes with the inhibition of histone demethylation including H3K36me2&3 and a disruption of normal transcriptional programs.

**Conclusions:**

Taken together, our results provide the first description of an association between global gene expression reprogramming and histone post-translational modifications during *P. falciparum* early sexual development. The stage II gametocyte-specific abundance of H3K36me2&3 manifests predominantly as an independent regulatory mechanism targeted towards genes that are repressed post-commitment. H3K36me2&3-associated repression of genes is therefore involved in key transcriptional shifts that accompany the transition from early gametocyte differentiation to intermediate development.

**Supplementary Information:**

The online version contains supplementary material available at 10.1186/s13072-021-00393-9.

## Background

Malaria remains a serious threat to public health in most of the developing world and is responsible for millions of deaths annually [1]. Nevertheless, progress is being made toward the global eradication of the disease. Malaria eradication relies on preventing the transmission of *Plasmodium* parasites between human hosts, facilitated by the mosquito vector. In the human host, malaria parasites exist either as the asexual proliferative stages, responsible for the symptoms of malaria, or as sexually differentiated, transmissible gametocytes [2, 3]. The extended 10- to 12-day process of gametocyte development is characterised by morphologically distinct stages (I–V) and is unique to the human malaria parasite, *Plasmodium falciparum.* Mature gametocytes are the only stage that can be transmitted by the mosquito vector and as such, the process of gametocytogenesis is an attractive target for the development of transmission-blocking strategies [4–6].

The stage transitions within the *P. falciparum* parasite life cycle are driven by global transcriptomic reprogramming that is tightly controlled by complex transcriptional and post-transcriptional regulation [7–9]. Additionally, epigenetic mechanisms are known to establish and maintain transcriptional programs that support asexual parasite proliferation and have been suggested to do the same during gametocytogenesis [10]. The histone post-translational modification (hPTM) landscape in *P. falciparum* parasites is dynamic with each life cycle stage characterised by a unique pattern of hPTMs, suggested to be foundational in establishing the specialised transcriptional program for the stage [11]. Accordingly, several hPTMs are confirmed to be functionally relevant for asexual parasite proliferation [12]. For example, H3K4me3, H3K8ac and H3K9ac are involved in the activation of stage-specific gene sets that are linked to proliferation and the maintenance of the euchromatic genome that is characteristic of asexual parasites [13–17]. By contrast, H3K36me2 has been proposed to be globally repressive in asexual parasites [18] and while more frequently associated with broad maintenance functions such as the conservation of genomic integrity, H3K36me2 has also been shown to direct transcriptional repression in other eukaryotes [19–21]. H3K36me3 is also associated with gene silencing and controls the expression of multi-gene families that encode invasion and exported proteins, thereby contributing to the parasite’s extensive capacity for phenotypic plasticity [22–24]. H3K36me2&3 are both well-documented regulators of eukaryotic cellular differentiation and development [19, 25, 26].

During proliferation, certain gametocyte-specific genes are silenced by their association with heterochromatin protein 1 (HP1) that is recruited by enrichment of H3K9me3 at these loci [27, 28]. Sexual differentiation requires the release of *ap2-g* (a member of the ApiAP2 transcription factor family, that is the master switch for gametocyte commitment [29]) from H3K9me3/HP1-mediated heterochromatin, resulting in a transcriptional environment that drives commitment to gametocytogenesis [29–32]. However, only a handful of studies have examined the functional relevance of epigenetic mechanisms in post-commitment gametocyte development [28, 30, 33]. Aside from the demonstrated role of H3K9me3/HP1 in heterochromatin rearrangement for stage-specific gene sets during gametocytogenesis [5], the function of hPTMs in generating and maintaining gametocyte-relevant transcriptional programs during sexual development have not been studied.

The dynamic nature of the hPTM landscape during gametocyte development and departure from the patterns observed during asexual proliferation suggest that hPTMs may be involved in stage-specific gene expression in gametocytes as has been demonstrated for asexual parasites [10, 12, 16, 28, 30, 34, 35]. Indeed, the concurrent shifts in the hPTM and transcriptomic landscapes associated with morphological transitions in gametocytes support this idea [11, 36]. Of particular interest to us was the striking peak abundance of H3K36me2&3 unique to stage II gametocytes that corresponds to the transcriptomic and morphological changes associated with the transition from early differentiation (stage I) to intermediate (stage II/III) development [11, 36]. We sought to interrogate the functional relevance of these hPTMs in gametocytogenesis [11]. To do so, we performed chromatin immunoprecipitation followed by high-throughput sequencing (ChIP-seq) on three distinct gametocyte populations and integrated the results with data from other ChIP-seq and gene expression profiling studies. We provide comprehensive genome-wide maps of the H3K36me2&3 occupancy during gametocytogenesis and show H3K36me2&3 enrichment in stage II gametocytes is involved in the transcriptional reprogramming underlying the transition from early gametocyte differentiation to intermediate development [36]. Additionally, we assessed the effects of chemically inhibiting histone demethylases (HDMs) on H3K36me2&3 levels and gene expression during early gametocyte development. Using histone methylation profiling and whole transcriptome analysis, we demonstrate the link between histone demethylation and the enhanced potency of the pan-selective Jumonji inhibitor, JIB-04 in gametocytes [37, 38]. This paper provides the first association between H3K36me2&3 and gene regulation in gametocytes and indicates that H3K36me2&3-associated gene repression is involved in key transcriptional shifts that accompany the transition from early gametocyte differentiation to intermediate development.

## Results

### H3K36me2&3 occupancy is dynamic in P. falciparum gametocytes

**Fig. 1:**
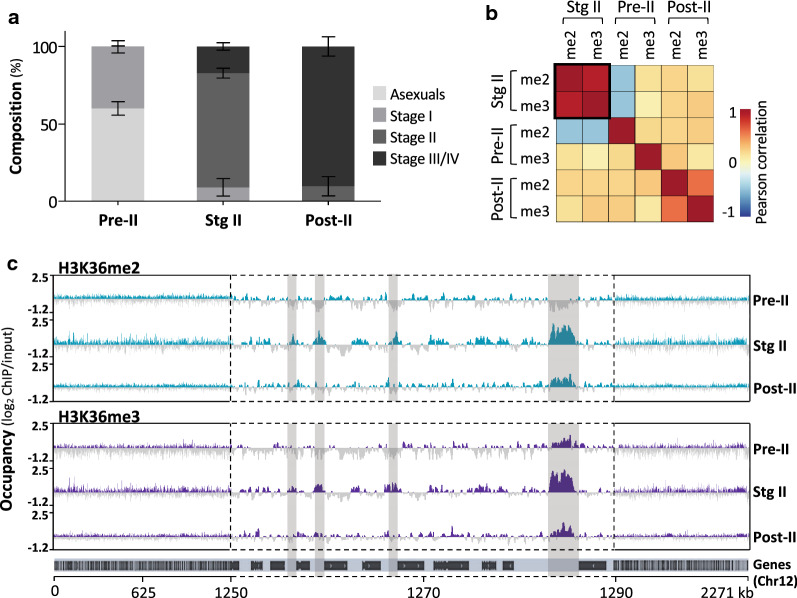
H3K36me2&3 have dynamic patterns of occupancy during *P. falciparum* early and intermediate gametocyte development. **a** The stage compositions (%) of gametocyte populations used for the ChIP-seq study, classified as pre-stage II (pre-II), stage II (stg II) and post-stage II (post-II) as determined microscopically. Data are from two independent biological repeats, ± S.E. **b** Plot of Pearson correlation coefficients for the genome-wide occupancy of H3K36me2&3 (me2 and me3) obtained for each gametocyte population with data representing two independent biological repeats. The black box highlights the increased positive correlation between H3K36me2&3 in stage II gametocytes. **c** H3K36me2&3 occupancy (average log_2_-transformed ChIP/input) over chromosome 12 for each gametocyte population. Data are representative of two independent biological replicates except for the pre-II gametocytes with a single replicate. Turquoise and purple represent regions where the hPTMs are present (log_2_ChIP/input ratios > 0) and dark grey represents regions of depletion (log_2_ChIP/input ratios ≤ 0). The light grey shadowed areas highlight stage II gametocyte-specific sites of H3K36me2&3 enrichment exemplified in a central region of the chromosome 12 (1250–1290 kb) with the positions of genes indicated below the occupancy tracks

To investigate the functional role of H3K36me2&3 in *P. falciparum* stage II gametocytes [11], we performed ChIP-seq on three distinct gametocyte populations spanning early gametocyte development and classified these as “pre-stage II”, “stage II” and “post-stage II” gametocytes. Gametocyte populations were morphologically binned into specific stages (Additional file 1: Figure S1A) resulting in a pre-stage II population (60 ± 4% asexual parasites; 40 ± 4% and 0.3 ± 0.4% stage I and II gametocytes, respectively) (Fig. 1a). The stage II population was enriched for stage II gametocytes (74 ± 3.2%, 9 ± 6% stage I and 17 ± 2.5% stage III gametocytes) without any asexual parasites present. Post-stage II samples consisted mostly of stage III/IV gametocytes (90 ± 6%; 10 ± 6% stage II gametocytes).

We performed ChIP-seq on these populations with ChIP-grade α-H3K36me2&3 antibodies with demonstrated specificity towards these histone marks from various organisms [39–43] as well as against *P. falciparum* [22, 44]. Nevertheless, we performed an independent validation of the selected antibodies with the results demonstrating specific detection of *P. falciparum* histone H3 with either H3K36me2 or H3K36me3 (Additional file 1: Figure S1B). ChIP-seq was performed on two independent biological replicates for each sample except for the pre-stage II gametocytes that only had one, with the resultant data for replicate samples well correlated (e.g. Pearson correlation, *r*^2^ = 0.93 and *r*^2^ = 0.94 between H3K36me2 biological replicates in stage II and post-stage II samples, respectively, Additional file 2: Table S1). We detected H3K36me2 and H3K36me3 individually in each of the sampled gametocyte populations and observed a strong positive correlation between the two hPTMs only in the stage II gametocytes (Pearson correlation, *r*^2^ = 0.9, Fig. 1b), and not the pre- or post-stage II gametocytes (Fig. 1b, Additional file 1: Figure S1C). Given that a single nucleosome would not contain di- and tri-methylated H3K36 concurrently, this likely reflects that one of these hPTMs is an intermediate of the other with our samples consisting of a combined pool of nucleosomes with either methylation state.

Our ChIP-seq results show that dynamic patterns of H3K36me2&3 is evident with “low–high–low” abundance profiles associated with pre-stage II, stage II and post-stage II gametocyte populations, respectively (Fig. 1c), as confirmed independently by ChIP-qPCR (Additional file 1: Figure S2). H3K36me2&3 is mostly absent in pre-stage II gametocytes and occupancy in stage II gametocytes is found preferentially in intergenic regions and upstream of gene coding sequences, and the levels of the hPTMs dissipate in post-stage II gametocytes (Fig. 1c). Given the unique abundance of H3K36me2&3 in the stage II gametocytes and the congruency of this with our previous proteomics data [11], our downstream analyses focussed on the preferential association of these hPTMs with stage II gametocytes.

### Stage II gametocytes have a unique pattern of H3K36me2&3 enrichment

**Fig. 2:**
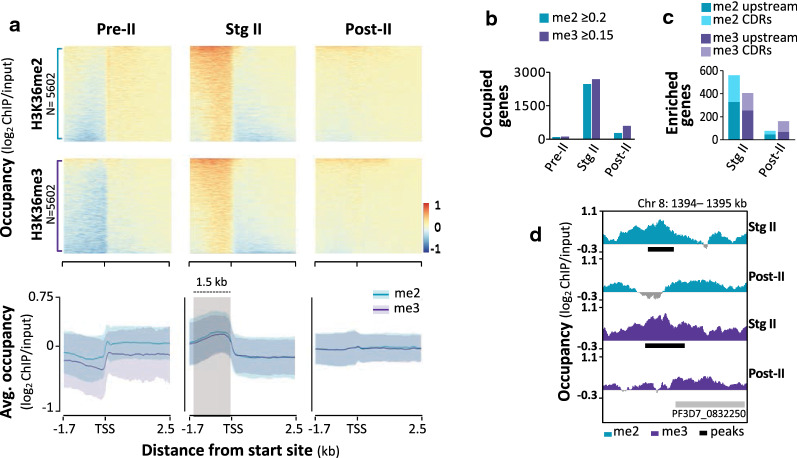
Stage II gametocytes have unique patterns of H3K36me2&3 occupancy and enrichment associated with genes. **a** H3K36me2&3 occupancy (average log_2_ ChIP/input binned into 50 bp regions) spanning 1.7 kb upstream and 2.5 kb downstream of transcriptional start sites (TSS) of 5206 *P. falciparum* genes (PlasmoDB genome annotation, v39) in pre-stage II (pre-II), stage II (stg II) and post-stage II (post-II) gametocyte populations. Data are representative of the average occupancy obtained for two independent biological repeats, except for pre-stage II gametocytes with only one. Genes were rank ordered individually in each heatmap. Summary plots of the H3K36me2&3 (turquoise and purple, respectively) occupancy in each of the gametocyte stages with ribbons representing ± SE. The light grey shadowed region indicates regions of increased occupancy in the stage II gametocytes. **b** Clustered column plots specifying the number of H3K36me2&3-enriched genes (i.e. log_2_ ChIP/input ≥ 0.2 and ≥ 0.15, respectively) with genes robustly enriched (i.e. log_2_ ChIP/input ≥ 0.5) represented by patterned bars. **C** Numbers of genes with H3K36me2&3 enrichment (MACS2 peaks, q-value < 0.05; present in both replicates) in stage II and post-stage II gametocytes. No peaks were detected for pre-stage II gametocytes. The proportion of genes with enrichment 1.5 kb upstream of TSSs is represented by the darker shade compared to the proportion of genes with enrichment in the coding regions (CDRs). **d** Log_2_-transformed ChIP/input ratio tracks for H3K36me2&3 in stage II and post-stage II gametocytes for a region on chromosome 8 to exemplify significant enrichment (MACS2 peaks present in both biological replicates, *q*-value < 0.05) in stage II gametocytes. Called peaks are indicated by the horizontal black bars

To determine the genome-wide positioning of H3K36me2&3, we interrogated the hPTM occupancy spanning each of *P. falciparum* genes for which sequencing data were obtained (Additional file 2: Table S2, Fig. 2a). H3K36me2&3 occupancy profiles of are distinctly uniform across the intergenic non-coding regions in stage II gametocytes, contrasting with more variable patterns in the pre- and post-stage II gametocytes where these marks are mostly depleted (Fig. 2a). In stage II gametocytes, H3K36me2&3 are concentrated 1.5 kb upstream of transcription start sites (TSSs, as defined previously [45]), with occupancy values (log_2_ChIP/input) peaking at > 0.2 and > 0.17, respectively, contrasting with a depletion of these hPTMs in coding regions (CDRs) (occupancy values of < 0.1 Fig. 2a).

The prominent abundance of H3K36me2&3 in stage II gametocytes translates to 2480 and 2691 genes occupied by H3K36me2 and -me3, respectively, with > 95% overlap present between these genes (i.e. above average log_2_ChIP/input value in stage II gametocytes, Fig. 2b). Within these genes, ~ 17% showed significant enrichment for H3K36me2&3 (significant MACS2 peaks, q-value < 0.05, present in both biological replicates) (Fig. 2c, Additional file 2: Table S3). For H3K36me2, 734 peaks were identified, corresponding to 327 genes with enrichment 1.5 kb upstream of TSSs and 233 genes with this hPTM enriched in CDRs (Fig. 2c). Similarly, H3K36me3 was identified at 569 sites across the genome in stage II gametocytes, equating to the enrichment upstream and in the CDRs of 252 and 154 genes, respectively (Fig. 2c). This enrichment is evident in stage II gametocytes only, with < 100 genes similarly enriched at each of these sites in post-stage II gametocytes (Fig. 2c) and none in pre-stage II gametocytes. The enrichment of H3K36me2&3 upstream of TSSs displays localised overlap in the presence of these marks (Fig. 2d). Since the presence of H3K36me2 as a transient intermediate in the formation of H3K36me3 has been reported elsewhere [22, 46–48] and cannot be excluded here, downstream analyses were performed on the combined set of enriched genes without distinguishing between the di- and tri-methylated state.

### H3K36me2&3 are associated with post-commitment transcriptional regulation in stage II gametocytes

**Fig. 3:**
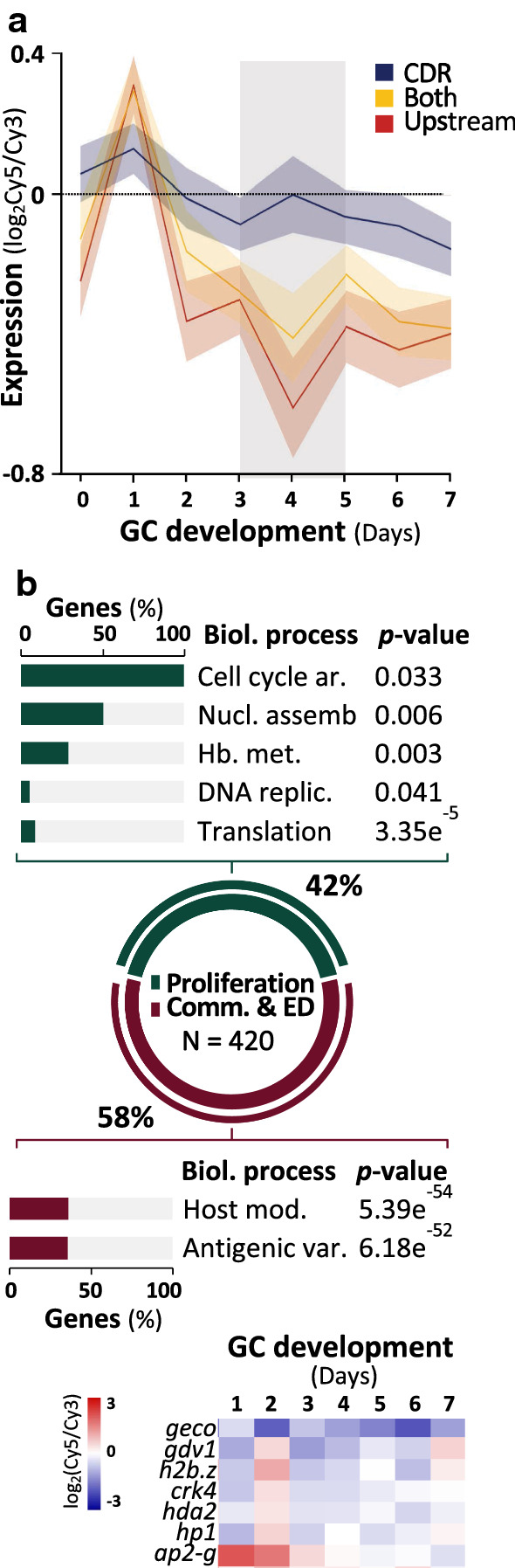
H3K36me2&3 are associated with transcriptional regulation in stage II gametocytes. **a** Average transcript abundance (log_2_Cy5/Cy3) on days 0–7 of gametocyte (GC) development [36] for genes with H3K36me2&3 enrichment in coding regions (CDR, blue), 1.5 kb upstream of TSSs (TSS, red) or both (gold) with ribbons representing ± S.D. They grey shadowed region highlights days on which H3K36me2&3-associated transcriptional changes are most prominent. **b** The 420 H3K36me2&3-enriched and repressed genes were grouped into those associated with proliferation (178 genes, green) that are significantly associated (*P* < 0 .05) with the biological processes indicated. The number of enriched genes is represented as a % of all background genes associated with the biological processes. Legend: ar. = arrest; nucl. = nucleosome; assemb. = assembly; replic. = replication; mod. = modification; var. = variation. Full descriptions of gene names and their PlasmoDB IDs are available in Additional file 2: Table S4

We categorised H3K36me2&3-enriched genes based on the location of the hPTMs (i.e. exclusively upstream of TSSs, exclusively in CDR or both) and compared these gene sets to their transcription profiles during gametocytogenesis, using a gene expression time-course dataset previously generated in our lab on the same clonal strain of *P. falciparum* NF54 parasites [34]. Corresponding expression profiles were obtained for days 2–6 of development (majority stage II gametocytes) for 92% of the H3K36me2&3-enriched genes (Additional file 2: Table S4). A clear variation in the expression profiles of the genes was present and associated with different sites of H3K36me2&3 enrichment. Only genes with H3K36me2&3 exclusively enriched in CDRs (37%) showed a slight increase in transcript abundance in stage II gametocytes (Fig. 3a), but is unclear if this increased abundance is relevant to gametocyte differentiation, as the level is not as prominent as in asexual parasites [22, 36].

The majority of H3K36me2&3-enriched genes (63%) are strongly repressed during stage II gametocytes, with the hPTMs present exclusively upstream of the TSSs in 342 of the 417 repressed genes (Fig. 3a). We find that 42% of these repressed genes are typically involved in processes associated with asexual proliferation, including those significantly associated with translation (*P* = 3.35e^−5^), haemoglobin metabolism (*P* = 0.003), cell cycle arrest (*P* = 0.033) and DNA replication (*P* = 0.041) (Fig. 3b, Additional file 2: Table S5). Furthermore, more than half of these are essential for asexual parasite development [49]. However, only 6% of these genes that are associated with H3K36me2&3 enrichment in asexual parasites [22], indicating that H3K36me2&3 enrichment in stage II gametocytes is targeted towards a distinct subset of asexual parasite-related genes that are specifically repressed in early gametocytogenesis.

We investigated the overlap of the H3K36me2&3-enriched genes in stage II gametocytes with genes previously described to be related to the processes of sexual commitment and early gametocyte differentiation [27, 30, 31, 36, 50–54]. A large proportion (58%, 242 genes) of the H3K36me2&3-enriched and repressed genes are linked to these processes (Fig. 3b, Additional file 2: Table S6). These include genes significantly associated with antigenic variation (*P* = 6.18e^−52^) and host cell modification processes (*P* = 5.39e^−54^, Fig. 3b, Additional file 2: Table S5), with a particularly strong repression seen for the *stevor* multi-gene family. H3K36me2&3-associated repression is present for early gametocyte markers (e.g. *geco,* PF3D7_1253000; *pfg14-744,* PF3D7_1477300 and *pfg14-748,* PF3D7_1477700) [51, 52, 55, 56] and key regulators of commitment and gametocytogenesis: *hp1* (PF3D7_1220900)*,* histone deacetylase 2 (*hda2*, PF3D7_1008000)*,* gametocyte development protein 1 (*gdv1*, PF3D7_0935400) and the ApiAP2 transcription factor, *ap2-g* (PF3D7_1222600, Fig. 3b) [28, 29, 53, 54, 57]. Sexual commitment is also associated with displacement of HP1 from 15 heterochromatic loci [30], and results in the expression of *ap2-g,* leading to the transcriptional cascade that drives sexual commitment [28, 29, 32, 57]. We therefore looked at the proportion of the H3K36me2&3-enriched and repressed genes in our dataset (242 genes involved in commitment) that are targets of AP2-G (31) or are transcribed following the displacement of HP1 [30]. We find that only 18% of the H3K36me2&3-enriched genes are activated by AP2-G or HP1 depletion during commitment, implying the H3K36me2&3-associated transcriptional repression of commitment-related genes in stage II gametocytes are irrespective of mechanisms by which they were activated in the preceding commitment steps. Importantly, 53% of the HP1-depleted genes that are expressed and drive commitment are enriched with these H3K36me2&3 in stage II gametocytes. As all of these genes remain depleted of HP1 [30], yet are associated with a sharp reduction in transcript abundance in the stage II gametocytes [36], their repression can be ascribed to their enrichment with H3K36me2&3. In the stage II gametocytes, H3K36me2&3 are therefore involved in the transcriptional repression of genes that drive commitment and early differentiation once their products become obsolete in post-commitment, developing gametocytes.

### H3K36me2&3-associated transcriptional repression is largely independent of other regulatory mechanisms

**Fig. 4:**
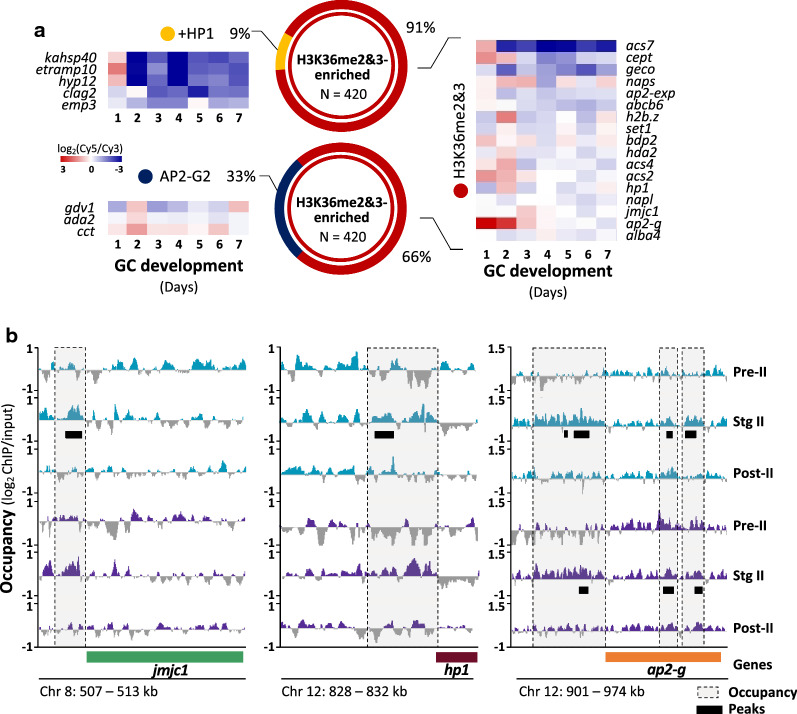
H3K36me2&3-associated transcriptional repression is largely independent of other regulatory mechanisms. **a** The H3K36me2&3-enriched and repressed gene set identified here for stage II gametocytes was compared to those previously determined to be repressed by HP1-mediated heterochromatin formation (+ HP1, gold) or by AP2-G2 (blue) in the stage II/III gametocytes. Certain genes from each of these categories are exemplified in the gene expression heatmaps [36]. **b** Log_2_-transformed ChIP/input ratio tracks of H3K36me2 (turquoise) and H3K36me3 (purple) occupancy for *jmjc1, hp1* and *ap2-g* in pre-stage II, stage II and post-stage II gametocytes, with the coding sequences of each represented by the green, maroon and orange blocks, respectively. Other genes are indicated by grey blocks. Dashed grey boxes represent regions of differential occupancy between the three stages and horizontal black bars indicate called peaks representing regions associated with H3K36me2&3 enrichment in stage II gametocytes. Full descriptions of gene names and their PlasmoDB IDs are available in Additional file 2: Table S4

Previous studies described other gene regulatory mechanisms that function during gametocytogenesis [30, 58], including HP1-mediated heterochromatin expansion throughout gametocyte development, resulting in the silencing of stage-specific gene sets [30]. Crucially, within our stage II gametocyte data, we identify that only 9% of the H3K36me2&3-enriched and repressed genes are also occupied by HP1 in stage II/III gametocytes (Fig. 4a), including those associated with knob-formation (e.g. *kahsp40,* PF3D7_0201800 and *emp3,* PF3D7_0201900) and protein export (e.g. *hyp).* These may therefore be regulated cooperatively by the two mechanisms. A second AP2 transcription factor, AP2-G2 is essential to gametocyte development beyond stage III and in asexual parasites, AP2-G2 represses a subset of genes through an association with H3K36me3 [58]. Only 33% of the H3K36me2&3-enriched genes in our data are also bound by AP2-G2 during intermediate gametocyte development (Fig. 4a), indicating that the transcriptional repression associated with the H3K36me3-AP2-G2 interaction also occurs for these genes in the early gametocytes. These include the genes encoding GDV1 that evicts HP1 from heterochromatic loci prior to commitment [53] and CCT (CTP:phosphocholine cytidylyltransferase, PF3D7_1316600), involved in the synthesis of PC from an alternative substrate under the lysoPC-depleted conditions that cue commitment [50, 59]. This indicates that the interaction between H3K36me2&3 and AP2-G2 may repress these important commitment-related genes once gametocytogenesis has been established.

However, the majority of the H3K36me2&3-enriched and repressed genes in stage II gametocytes are not associated with any other regulatory mechanisms and is likely regulated by a novel repressive mechanism involving the stage II gametocyte-specific enrichment of H3K36me2&3. This includes lysoPC synthesis-associated genes (e.g. *acs2,* PF3D7_0301000: *acs4,* PF3D7_1372400; *acs7* PF3D7_1200700 and *cept,* PF3D7_0628300) and several epigenetic and transcriptional regulators (e.g. *hda2; set1,* PF3D7_0629700 and *alba4,* PF3D7_1347500; Fig. 4a). Importantly, *ap2-g, hp1* and a Jumonji lysine demethylase (KDM) *jmjc1* (PF3D7_0809900, Fig. 4a), are enriched for H3K36me2&3 upstream of TSSs in stage II gametocytes, but not in pre- and post-stage II gametocytes (Fig. 4b), with only *ap2-g* additionally having enrichment within the CDR. This enrichment of these regulatory proteins with H3K36me2&3 provides the first insights into the mechanism by which these genes are “switched off” after commitment to allow gametocyte development to proceed. Furthermore, the H3K36me2&3 enrichment and repression of *jmjc1* is of interest given its homology with the H3K36-specific, lysine demethylase 2 (*kdm2*) family [44]. As such, the repression of *jmjc1* may be present as means of safeguarding the integrity of H3K36me2&3 enrichment and the associated transcriptional repression in the stage II gametocytes. Taken together, these results implicate H3K36me2&3 as a novel mechanism of transcriptional repression in stage II gametocytes that is largely independent of other mechanisms described to date.

### Inhibition of H3K36 demethylation by JIB-04 is associated with altered patterns of transcription in P. falciparum gametocytes

**Fig. 5:**
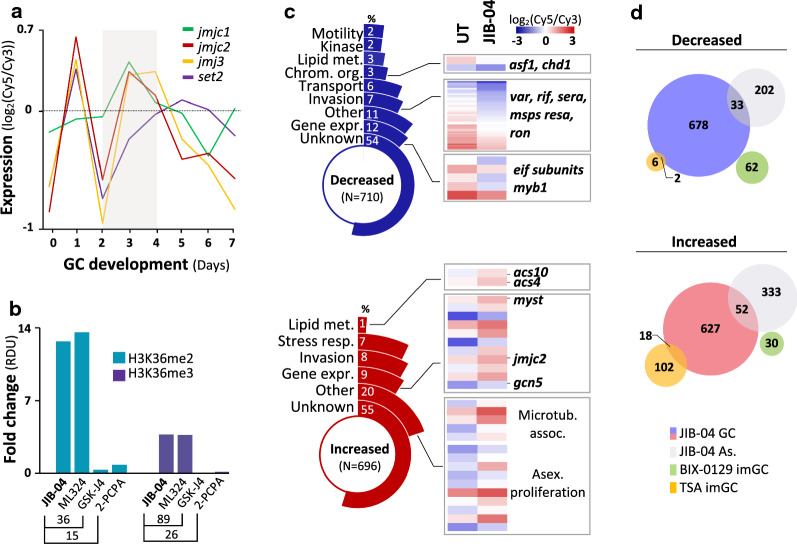
Chemical inhibition of histone demethylase activity leads to the disruption of normal gene expression in gametocytes** a** Expression profiles (log_2_Cy5/Cy3) of genes encoding the *P. falciparum* Jumonji-C domain-containing demethylases (*jmjc1, jmjc2* and *jmj3)* and the H3K36-specific methyltransferase, *set2* on days 0 to 7 of gametocyte (GC) development [36], with the grey shadowed region representing days of increased transcript abundance of the demethylases that are associated with stage II gametocytes. **b** H3K36me2&3 abundance on day 4 of gametocyte development following treatment (24 h, 5 µM) with the Jumonji demethylase inhibitors JIB-04, ML324 or GSK-J4 or the lysine specific demethylase 1 (LSD1) inhibitor 2-PCPA expressed as fold changes (treated/parallel untreated control gametocytes) of relative density units (RDU). **c** Functional classification of differentially expressed genes (log_2_ fold change ≥ 0.5 in either direction) with decreased (blue) or increased (red) transcript abundance in response to the inhibition of histone demethylase activity by JIB-04. The % of differentially expressed genes assigned to each biological category is shown. The proportion (%) of differentially expressed genes s that had H3K36me2/3 occupancy (light grey) or enrichment (dark grey) in the stage II gametocytes (data representative of two independent biological repeats) are shown on the two inner most segments. Transcript levels in the untreated and JIB-04 treated gametocytes are exemplified for certain differentially expressed genes. **d** Comparison of differentially expressed genes (blue and pink) in gametocytes (GC) treated with JIB-04 in this study with differentially expressed genes in JIB-04 treated asexual parasites (shown in grey, [38]), immature gametocytes (imGC) treated with the G9a-specific inhibitor BIX-0129 [69] and imGC treated with the HDAC inhibitor Trichostatin A [70]. Full descriptions of gene names and their PlasmoDB IDs are available in Additional file 2: Table S4

To investigate the functional relevance of enzymes involved in deposition (histone methyltransferases, HMT) and removal (KDMs) of H3K36me2&3, we interrogated inhibition of selected enzymes on H3K36 methylation levels on days associated with stage II gametocyte development. In asexual parasites, the HMT SET2 (PF3D7_1322100) methylates H3K36 [44, 60], but is comparatively poorly expressed during gametocytogenesis (Fig. 5a). Although knockout lines of *set2* are available [22, 60], these do not produce gametocytes and therefore could not be used to interrogate the involvement of SET2 in H3K36 methylation stage II gametocytes. Three Jumonji KDMs of *P. falciparum* (*jmjc1* (PF3D7_0809900)*, jmjc2 (*PF3D7_0602800) and *jmj3* (PF3D7_1122200)) are transcribed in stage II gametocyte development, strongly suggesting that at least one of these enzymes demethylates H3K36 (Fig. 5a). We used inhibitors of the KDMs and could show that JIB-04 (pan-selective) and ML324 (targeting KDM4, *Pf*JMJ3), both with activity against gametocytes [38, 61, 62], caused hypermethylation of H3K36 (~ 13-fold, Fig. 5b, Figure S3A). This was not seen for GSK-J4 (inhibiting KDM6, H3K27me3 selective) or PCPA-2 (targeting LSD1) (Fig. 5b) [63, 64]. This indicates that one of the *P. falciparum* Jumonji KDMs demethylate H3K36me2&3 after they peak in abundance in stage II gametocytes.

We were particularly interested in the transcriptional effects of JIB-04 in gametocytes. As such, we performed genome-wide transcriptional profiling of stage II gametocytes treated with JIB-04 for 24 h (Additional file 3: Table S7). JIB-04-treated gametocytes displayed differential expression of ~ 13% of the genome (711 and 696 decreased and increased abundance, respectively, Fig. 5c, Additional file 1: Figure S3B), similar to the restricted effect induced by this inhibitor in asexual parasites [38]. Differentially expressed genes with decreased transcript abundance are associated with various processes including chromatin organisation (3%), gene expression (12%) and transport (6%), each of which have confirmed roles in gametocyte differentiation and development [30, 36, 51, 53–55, 65–67] (Fig. 5c), but are also seen in asexual parasites [38] (Fig. 5d). Interestingly, we also find an increase in the transcript levels of *jmjc2* and the histone acetyltransferase-encoding genes, *gcn5* (PF3D7_0823300) and *myst* (PF3D7_1118600) (Fig. 5c) [14, 68], which may reflect an attempt to counteract the abnormal histone methylation patterns induced by JIB-04. While JIB-04-treated gametocytes do not share common differentially expressed genes with the G9a-specific HMT inhibitor, BIX-01294 [69], some overlap is present with HDAC inhibition by trichostatin A [70]. This is in line with other studies which showed similarities in the gene expression signatures of JIB-04 and TSA in cancer cell lines [71].

Of the genes differentially expressed due to JMJ inhibition, 25% are also enriched for H3K36me2&3 and whilst this confirms the involvement in JMJ activity and removal of H3K36 methylation marks, we cannot exclude that other marks, e.g. H3K9me is not also involved. Indeed, the use of this general Jumonji HDM inhibitor highlights the importance of normal histone methylation patterns for transcriptional reprogramming during gametocyte differentiation and development that involves H3K36 methylation.

## Discussion

Understanding the gene regulatory mechanisms that drive differentiation and development in *P. falciparum* gametocytes is essential for the discovery and advancement of novel malaria transmission-blocking strategies [72]. Here, we indicate a dynamic yet stage-specific nature of H3K36me2&3 enrichment in stage II gametocytes, associated transcriptional reprogramming occurring post-commitment that is required to drive gametocyte development in *P. falciparum*.

Our approach allowed for the delineation of the genomic regions dynamically occupied by H3K36me2&3 during gametocytogenesis. We demonstrate that the location-dependent outcome of H3K36me2&3 deposition to either intergenic regions or CDRs in regulating gene expression is retained in *P. falciparum* gametocytes. The stage II gametocyte-specific abundance of H3K36me2&3 manifests as a wide-spread, yet largely intergenic enrichment that results chiefly in transcriptional repression, congruent with the characteristic patterns of these hPTMs when functioning repressively in other organisms, particularly during cellular differentiation [73–81]. CDR enrichment is associated with transcriptionally permissive genes, similar to other eukaryotes [22, 82, 83]. The distribution of H3K36me2&3 enrichment and the associated transcriptional effects in stage II gametocytes aligns with the SET2-mediated deposition of H3K36me2&3 in the CDRs of active *var* genes in asexual parasites, while enrichment of this hPTM upstream of the TSSs silences these genes [22, 60]. These similar patterns of H3K36me2&3-associated transcriptional repression in stage II gametocytes and presence of transcript levels for *set2* during early development [36], suggests this HMT is the most likely candidate enzyme of H3K36 methylation in gametocytes. The proposed shared regulatory mechanisms of H3K36 methylation between asexual parasites and gametocytes are supported by similar hypermethylation of this PTM in both asexual parasites [38] and here in gametocytes, by either pan-reactive JMJ inhibitors (like JIB-04) or even with the JMJC2-specific inhibitor, ML324.

Accumulating evidence demonstrates that epigenetic regulators are essential drivers of the transcriptional reprogramming necessary for cellular differentiation [73, 84–86]. Accordingly, for *P. falciparum* gametocyte differentiation, we indicate a role of H3K36me2&3 in governing transcriptional shifts that coincide with the transition from early differentiation to intermediate gametocyte development. The H3K36me2&3-associated repression of genes involved in asexual proliferation-specific processes (e.g. DNA replication), does indeed attest to the influential role of H3K36me2&3 at this transition point. Additionally, the enrichment of genes that are upregulated under conditions of lysoPC depletion links H3K36me2&3 with the parasite’s earliest responses to the environmental cue for sexual commitment [50]. Specifically, we distinguish an exclusive association between H3K36me2&3 and the post-commitment repression identified previously for *hp1* and *ap2-g* [36, 87] and as such, provide the first insights into the mechanisms that govern the regulation of these genes in differentiated gametocytes [36, 87]. While we find that H3K36me2&3 function largely independently of other regulatory mechanisms, we do find an association between these hPTMs and AP2-G2 regulation in gametocytes as reported previously for asexual parasites [58] as well as a link between H3K36me2&3 and the formation of heterochromatin in early gametocytes in accordance with similar observations in other organisms [75, 88, 89].

## Conclusions

This work highlights the crucial nature of the epigenetic mechanisms underlying the vast transcriptional reprogramming associated with *P. falciparum* gametocyte differentiation [11, 30, 35, 36]. Here, we demonstrated an association between H3K36me2&3 and the repression of proliferation- and commitment-specific transcripts once they become obsolete in differentiated gametocytes. This marks H3K36me2&3 as important hallmarks of a transition point in gametocyte development from early differentiation to intermediate development. We conclude that the transcriptional repression associated with H3K36me2&3 enrichment occurs largely independent of other mechanisms described to date for *P. falciparum* parasites. Importantly, we identify an exclusive association between H3K36me2&3 enrichment and *hp1* and *ap2*-g (25, 31), thereby providing the first insights into the mechanisms governing the regulation of these genes in the post-commitment, terminally differentiated gametocytes. The findings of this study make substantial contributions to the understanding of epigenetics in *P. falciparum* gametocytes which until recently had remained largely unexplored.

## Methods

### Parasite culturing

*P. falciparum* NF54 asexual parasite cultures were maintained in vitro at 37 °C in human erythrocytes at a 5% haematocrit (ethics approval obtained from the University of Pretoria Research Ethics Committee, Health Sciences Faculty 506/2018) and synchronised using 5% D-sorbitol as previously described [90, 91]. *P. falciparum* gametocytes were induced as previously described [92–94] through a combination of nutrient starvation and a drop in haematocrit from synchronous (≥ 95% ring stages) asexual parasite cultures (0.5% parasitaemia, 6% haematocrit). Media (RPMI-1640, 23.8 mM Na_2_CO_3_, 0.024 mg/ml gentamycin, 25 mM HEPES, 0.2 mM hypoxanthine and 0.5% (w/v) Albumax II) was replaced 48 h after initiation. The haematocrit was reduced to 4% 72 h after initiation (hereafter referred to as day 0) and maintained as such throughout gametocyte development with daily replacement of glucose-enriched (20 mM) media. Residual asexual parasites were removed by supplementing glucose-enriched media with 50 mM N-acetylglucosamine (Sigma-Aldrich) from day 1 of development. Gametocytes were sampled on days 2, 4 and 7 post-induction as the pre-stage II, stage II and post-stage II samples, respectively. Two independent cultures were used as biological replicates for each ChIP-seq and ChIP-qPCR experiment. Parasite proliferation and gametocyte differentiation were monitored daily by microscopic evaluation of Giemsa-stained smears.

### Antibody validation

The specificity of commercially obtained ChIP-grade rabbit anti-H3K36me2 (Abcam, ab9049) and anti-H3K36me3 (Abcam, ab9050) antibodies (same batch numbers as previously validated for specificity against H3K36me2&3, [22, 34, 39–41, 43]) was tested using modified and unmodified synthetic peptides (Genscript) of the *P. falciparum* 3D7 histone H3 sequence as per ENCODE standards (www.encodeproject.org; Additional file 4: Table S8). Modified peptides were di- or tri-methylated on K9 or K36 each with a corresponding unmodified K9 (ARTKQTARKSTAGKAPRKQ) and K36 (ARKSAPISAGIKKPHRYRPG) peptide. Nitrocellulose membranes spotted with 25 ng of each peptide were blocked for 30 min in blocking buffer (5% milk powder in TBS-t (50 mM Tris (pH 7.5), 150 mM NaCl and 0.1% (v/v) tween-20) and incubated with primary antibodies (1:5000) against H3K36me2 or H3K36me3 overnight followed 1 h incubation with secondary antibody (1:10,000 goat anti-rabbit IgG conjugated to HRP). Chemiluminescent signal (Pierce Super Signal West Pico PLUS Chemiluminescent Substrate) was quantified by densitometry using ImageJ analysis software [95].

### Chromatin immunoprecipitation

ChIP was performed as previously described [31] with modifications. Gametocytes (1–3% gametocytaemia, fixed in 1% formaldehyde for 10 min at 37 °C followed by 0.125 mM glycine quenching) were released from erythrocytes using 0.1% (w/v) saponin. Thereafter, the gametocytes were resuspended in lysis buffer (10 mM Hepes (pH 7.9), 10 mM KCl, 0.1 mM EDTA (pH 8.0), 0.1 mM EGTA (pH 8.0)) and lysed with 0.25% Nonidet P-40 and douncing. Nuclei were then resuspended in Covaris shearing buffer (0.1% sodium dodecyl sulfate (SDS), 10 mM Tris (pH 8.1), 1 mM EDTA) and sonicated with the conditions: 5% duty cycle, 75 W peak incident power, 200 cycles per burst for a total treatment time of 300 s using a M220 ultrasonicator (Covaris). The sonicated chromatin was then pre-cleared using Protein A/G magnetic beads (Millipore 16–663) and aside from a small quantity kept separately as input material, chromatin was incubated overnight with 3 µg of anti-H3K36me2 or anti-H3K36me3. Reversal of cross-linking was achieved by adding 0.2 M NaCl followed by RNAseA and ProteinaseK treatment. DNA was purified using the Qiagen MinElute kit and used to prepare DNA libraries for sequencing and qPCR validation.

### Library preparation and sequencing

DNA libraries were prepared for sequencing as previously described [31]. End-repair and A-tailing of DNA fragments were achieved using NEBNext End Repair Enzyme Mix (#E6051A) and 3′-5′ exo Klenow fragment (#E6054A). Indexed adaptors (NEXTflex DNA-Seq barcoded adaptors) were ligated (NEB Quick Ligase, #M2200L) to fragments that were then size selected (250 bp) using 0.7 × AMPure XP beads (Beckman Coulter). The selected fragments were amplified using KAPA HiFi with dNTPs, and NEXTflex primer mix (#NOVA-514107–96). The PCR products were purified (0.9 × AMPure XP beads) and quantified using the Qubit fluorometer HS DNA kit. Sequencing was carried out on an Illumina HiSeq 2500.

### ChIP-qPCR

ChIP-qPCR was performed on the same material used for ChIP-seq with primers listed in Additional file 4: Table S9. Using serial dilutions of 3D7 genomic DNA all primers were determined to be > 90% efficient and specific, evidenced by single peaks on the melting curves. ChIP-qPCR data were obtained using the Applied Biosystems 7500 Real-Time PCR machine and SDS v1.4 and analysed using the ΔΔCt method. Values are expressed as fold enrichment of immunoprecipitated to input DNA, averaged for two biological replicates for H3K36me2&3 in stage II and post-stage II gametocytes. Pre-stage II gametocyte data represents only 1 replicate as insufficient DNA was obtained for the second replicate samples.

### Detection of changes in histone methylation

Gametocytes were treated with JIB-04 (5 µM) on days 2, 3 and 4 of development and sampled 24 h later. Histones were extracted as described before [17] with minor modifications. Nuclei were extracted by Dounce homogenisation in hypotonic lysis buffer (10 mM Tris–HCl (pH 8), 0.25 M sucrose, 3 mM MgCl_2_, 0.2% (v/v) Nonidet P-40) and protease inhibitors (Roche)). Histones were isolated from nuclei resuspended in Tris buffer (10 mM Tris (pH 8.0), 0.8 M NaCl, 1 mM EDTA and protease inhibitors) by overnight acid-extraction (0.25 M HCl, with rotation at 4 °C) and subsequent precipitated with 20% TCA. Histone pellets were washed with acetone, reconstituted in dddH_2_O and spotted quantitatively (100 ng per sample) on nitrocellulose membranes. Membranes were submerged in blocking buffer for 30 min followed by 1 h incubation with α-H3K36me2 (Abcam, ab9049) or α-H3K36me3 (Abcam, ab9050) primary antibody dilutions (1:5000 in TBS-t). Membranes were washed three times in TBS-t and then incubated with goat α-rabbit IgG antibody conjugated to HRP (1:10000) for 1 h. Chemiluminescent signal (Pierce SuperSignal West Pico PLUS Chemiluminescent Substrate) was quantified with ImageJ analysis software [95].

### DNA microarrays

DNA microarrays (60-mer, Agilent Technologies, USA) based on the full *P. falciparum* genome as previously described [96] were used to assess global transcriptomic changes in gametocytes treated with JIB-04. Day 2 and day 3 gametocyte cultures (1–3% gametocytaemia, 4% haematocrit) were treated with 5 µM JIB-04 (Cayman Chemicals) for 24 h followed by isolation of gametocytes using 0.01% (w/v) saponin. Total RNA was isolated with a combination of TRIzol (Sigma-Aldrich, USA) and phenol–chloroform extraction and subsequently used to synthesise cDNA as previously described [96] for the untreated and JIB-04 treated day 2 and day 3 gametocyte samples. Sample cDNA was labelled with Cy5 dye (GE Healthcare, USA) prior to hybridisation to arrays with an equal amount (350–500 ng) of Cy3-labelled (GE Healthcare, USA) reference pool containing equal amounts of cDNA from each gametocyte sample and mixed stage 3D7 asexual parasites. After hybridisation, the slides were scanned on a G2600D (Agilent Technologies, USA) scanner and normalised signal intensities for each oligo were extracted using the GE2_1100_Jul11_no_spikein protocol and Agilent Feature Extractor Software (v 11.5.1.1) as described before [96].

### Data analysis: ChIP-seq

ChIP-seq data analysis was performed according to ENCODE guidelines [97] and as described previously [31, 58]. Sequence read quality was determined using FastQC [98] prior to analysis and adapter sequences were removed using Trimmomatic (v0.32.3) [99]. Reads were mapped to the *P. falciparum* 3D7 genome (v39 obtained from PlasmoDB) and duplicate and low-quality reads filtered using BWA-MEM (v0.4.1) [100] and SAMtools (v1.1.2) [101], respectively. Correlations between corresponding biological replicates were determined prior to subsequent analysis. The deepTools suite (v3.1.2.0.0) [102] was used to plot the average occupancy of hPTMs (plotProfile and plotHeatmap tools) and to generate bigwig files that were viewed in IGV [103]. Genome-wide occupancy was calculated and is reported as log_2_-transformed ChIP/input ratios averaged for 1 kb bins. Occupancy 1.5 kb upstream of gene TSSs (TSSs as defined by Adjalley et al*.,* 2016 [45]; PlasmoDB, genome release v39) and within the CDR was calculated from log_2_ChIP/input ratios over 50 bp bins averaged across these regions. MACS2 [104] was used with the broad peak option (q-value < 0.05; ‘-broad—cutoff 0.1′) to call peaks as described by others [31, 58, 105]. As two biological replicates were used for each experiment, only regions enriched in both biological replicates were used for all subsequent analyses. These were identified and annotated using the Multiple Intersect function and ClosestBED, respectively, from the BEDtools suite [106]. All results are representative of data averaged for two biological replicates unless otherwise stated. GO enrichment analyses were performed with PlasmoDB using a *P*-value cut-off ≤ 0.05.

### Data analysis: DNA microarrays

Microarray signal intensities that passed Genepix standard background filters (*P* < 0.01), were normalised with Robust-spline for within-array and G-quantile for between slide normalisation in R (v3.2.3, www.r-project.org) using the limma and marray packages. The fit of a linear model was used to obtain log_2_-transformed expression values (log_2_Cy5/Cy3). Genes with log_2_-transformed FC ≥ 0.5 in either direction were defined as differentially expressed in the JIB-04-treated gametocytes. Visualisation of differentially expressed genes was performed using TIGR MeV with functional classification of genes to significantly associated biological processes (*P*-values ≤ 0.05) performed using PlasmoDB (v39, www.plasmodb.org).

## Supplementary Information

**Additional file 1. **Figures and corresponding legends for **Figures S1–S3** as a supplement to main text and figure.

**Additional file 2: Table S1.** Pearson correlation of genome-wide read counts binned into 1 kb for corresponding biological replicates used for ChIP-seq. **Table S2.** Log_2_-transformed ChIP/input values for H3K36me2&3 occupancy upstream of 5602 *P. falciparum* genes in pre-stage II, stage II and post-stage II gametocytes. **Table S3.** Sites of H3K36me2&3 enrichment that were present in both biological replicates as determined by peak calling for stage II gametocytes. **Table S4.** Expression values on days 0 to 10 of gametocyte development for the gene nearest to each significant peak. **Table S5.** Gene ontology analysis results for biological processes significantly associated with H3K36me2&3-enriched gene sets in stage II gametocytes. **Table S6.** H3K36me2&3-enriched genes in stage II gametocytes previously identified as commitment- or early differentiation-related or associated with HP1, AP2-G2 or AP2-G2 in other studies [27, 30, 31, 36, 50–54, 58].

**Additional file 3: Table S7.** Gene expression values and log_2_fold changes from microarray experiments for the untreated and JIB-04 treated gametocytes on day 2 and 3 with associated functional categories for each gene.

**Additional file 4: Table S8.** Sequence information of peptides used for antibody validation. **Table S9.** Primer sequences used in ChIP-qPCR validation.

## Data Availability

The ChIP-seq datasets generated and supporting the conclusions of this article are deposited in the NCBI Sequence Read Archive, accession number GSE163432 available at https://www.ncbi.nlm.nih.gov/geo/query/acc.cgi?acc=GSE163432. DNA microarray data are available from the Gene Expression Omnibus repository, accession number GSE163189, https://www.ncbi.nlm.nih.gov/geo/query/acc.cgi?acc=GSE163189. Microarray time-course data [36] were obtained from the Gene Expression Omnibus (GEO) under the accession number GSE104889 and presented as expression values (log_2_Cy5/Cy3) in heatmaps generated using TIGR MeV. Additional data sets pertaining to H3K9me3/HP1 in early gametocytes (GSE102695) [30], AP2-G binding sites (GSE120448 and SRP091939) [31] and AP2-G2 binding sites [58] were obtained from GEO and SRA [30, 31, 35]. The panel of commitment- and development-specific genes was compiled from supplementary data files associated with previous publications [27, 30, 31, 36, 50–54]. Data pertaining to the transcriptome analysis of JIB-04 in asexual parasites, BIX-01294 and TSA were obtained from the supplementary files associated with [38, 69, 70].

## Abbreviations

CDR: Coding region
ChIP-seq: Chromatin immunoprecipitation followed by sequencing
FC: Fold change
GO: Gene ontology
H3K36me2&3: Histone 3 lysine 36 di- and tri-methylation
HAT: Histone acetyltransferase
HDAC: Histone deacetylase
HMT: Histone methyltransferase
HP1: Heterochromatin protein 1
hPTM: Histone post-translational modification
JMJC: Jumonji-C
KDM: Lysine demethylase
LSD1: Lysine-specific demethylase 1
lysoPC: Lysophosphatidylcholine
PC: Phosphatidylcholine
TSS: Transcription start site
