# Supplementary material for: H3K36 methylation reprograms gene expression to drive early gametocyte development in Plasmodium falciparum
Source: Epigenetics Chromatin. 2021 Apr 1;14:19. doi: 10.1186/s13072-021-00393-9 (PMC8017609; doi:10.1186/s13072-021-00393-9)
Supplement: Supplementary file 1 — Additional file 1. Figures and corresponding legends for Figures S1–S3 as a supplement to main text and figure. [file 13072_2021_393_MOESM1_ESM.pdf]

H3K36 methylation reprograms gene expression to drive early gametocyte development in Plasmodium falciparum

Jessica Connacher[1], Gabrielle A. Josling[2], Lindsey M. Orchard[2], Janette Reader[1], Manuel Llinás[2,3], Lyn-Marié Birkholtz[1*]

SUPPLEMENTARY FIGURES

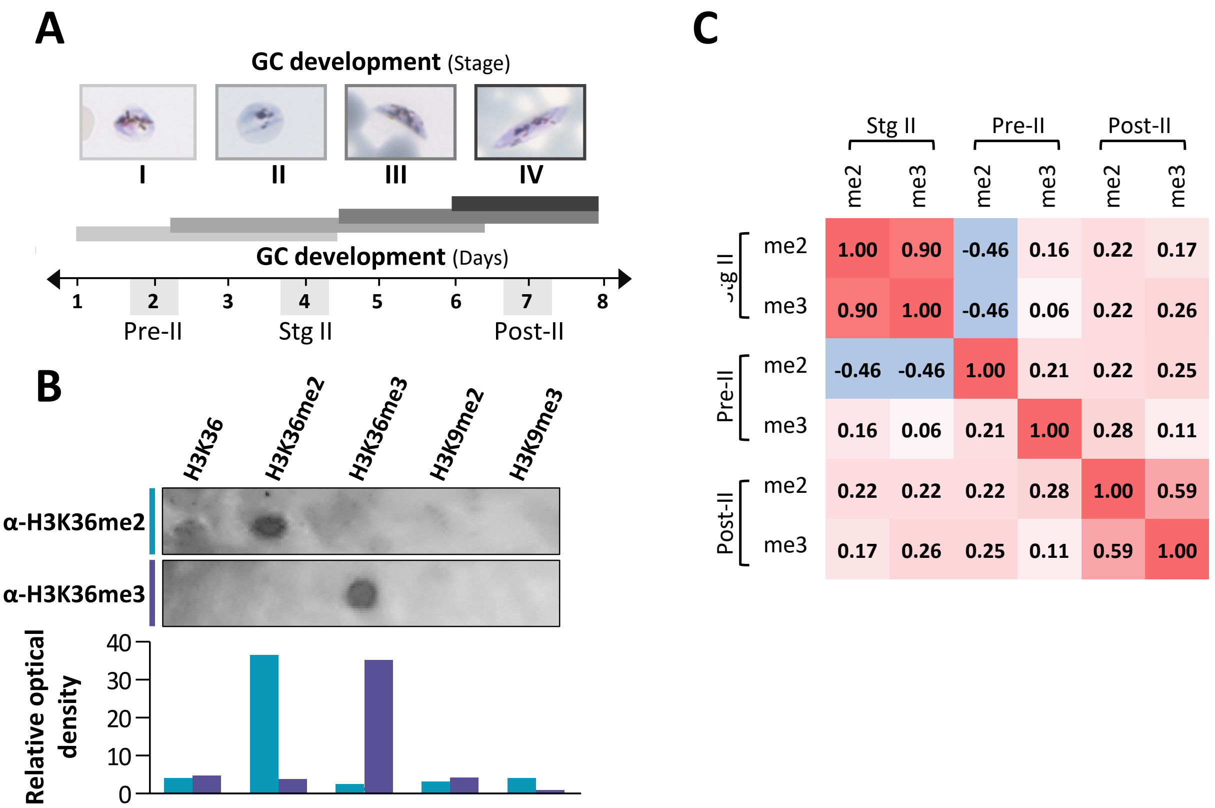

Figure S1. Gametocyte sampling strategy, anti-H3K36me2&3 antibody validation and correlation analysis of ChIP-seq samples. A Microscopic evaluation of Giemsa-stained gametocytes was used to determine the composition of pre-stage II (pre-II), stage II (stg II) and post-stage II (post-II) gametocyte samples isolated on days 2, 4 and 7 of gametocyte (GC) development, respectively. **B** Validation of α-H3K36me2&3 antibodies to specifically detect *P. falciparum* histone H3 with the respective modification (either H3K36me2 or H3K36me3) by dot blot analysis using unmodified (H3K36 and H3K9) and modified (H3K36me2, H3K36me3, H3K9me2 and H3K9me3) synthetic histone peptides. Peptide sequence information is provided in Additional file 4: Table S8. Relative optical density was calculated to quantify the degree to which the antibodies detect each peptide. The H3K36me2&3 antibodies had 9- and 7.5-fold greater specificity towards their corresponding modified peptides compared to the unmodified peptides, respectively **C** Pearson correlation coefficients for H3K36me2 (me2) and H3K36me3 (me3) occupancy (average $\log_2$ChIP/input ratio across the genome, binned into 1 kb regions, for two biological replicates*) in pre-II, stg II and post-II gametocytes. *H3K36me3 in pre-stg II had only one biological replicate.

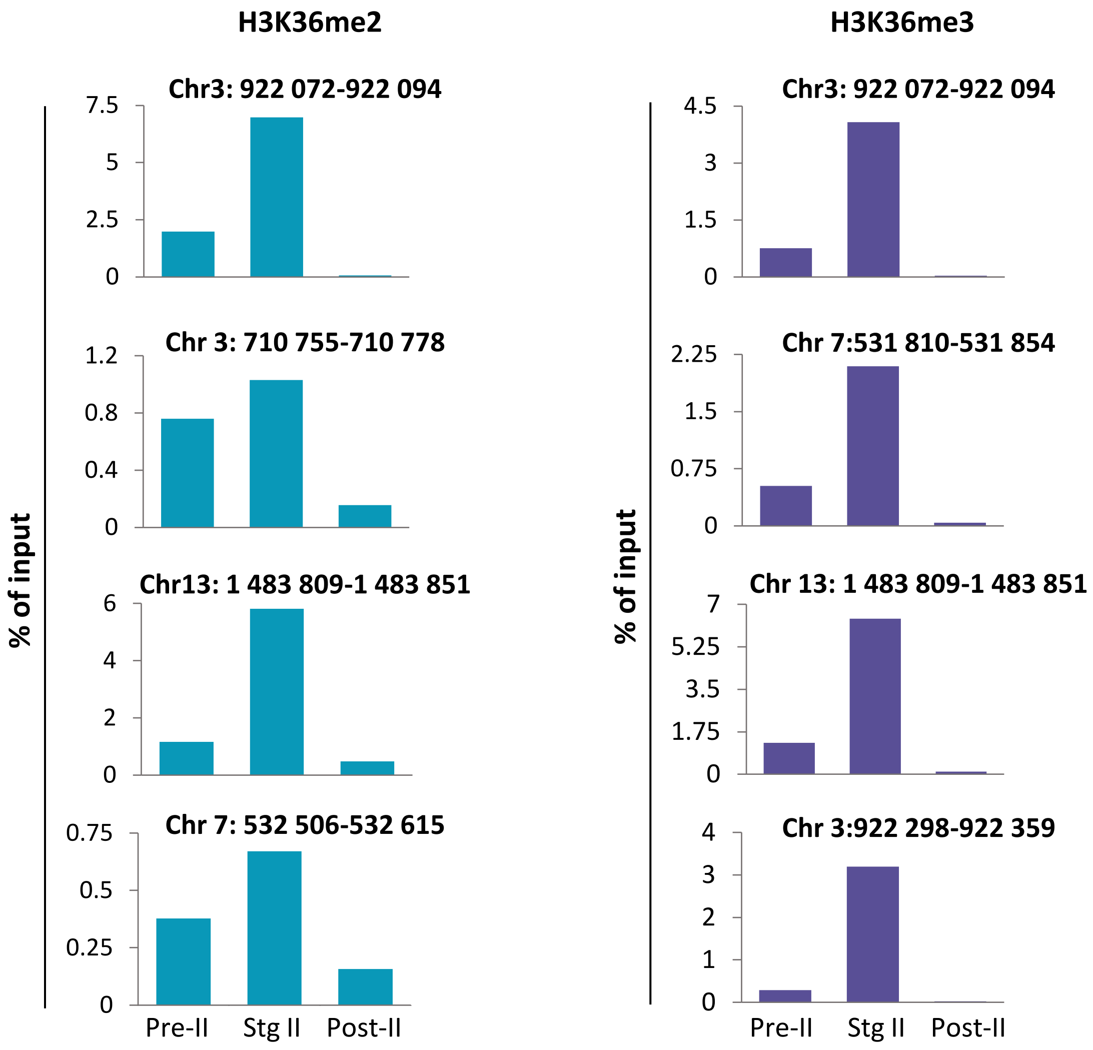

Figure S2. ChIP-qPCR validation of stage-specific enrichment of H3K36me2&3. ChIP-qPCR results for H3K36me2&3 occupancy in pre-II, stg II and post-II gametocytes expressed as a % of input with the region of the chromosome amplified by the primers indicated above each graph. The sequence information of each primer pair is provided in Additional file 4: Table S9

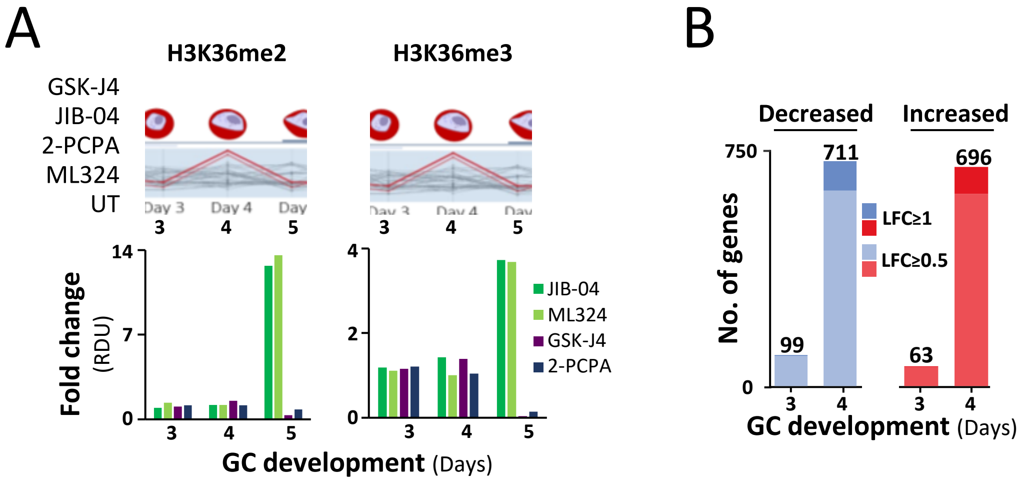

Figure S3. Histone demethylase inhibitors alter H3K36 methylation status and gene expression patterns in *P. falciparum* early gametocytes. A Detection of changes in H3K36me2&3 abundance using dot blot analysis of day 3-5 of gametocytes treated with the Jumonji demethylase inhibitors GSK-J4, JIB-04 or ML324 or the lysine specific demethylase 1 (LSD1) inhibitor 2-PCPA for 24 h (5 µM) compared with parallel untreated (UT) control gametocyte populations. Results of densitometry analysis are shown as fold changes (treated/untreated) of the relative density units (RDU). **B** Comparison of the proportions of differentially expressed genes (log_2FC (LFC) ≥0.5 in either direction) on days 3 or 4 of gametocyte development following treatment with JIB-04 (24 h, 5 µM) with the total numbers of these differentially expressed genes shown at the top of each bar. Blue and red represent the proportions of genes with decreased and increased transcript abundance, respectively, with the darker shading indicating genes with LFC ≥1.
